# Quinoa (*Chenopodium quinoa* Willd.) supplemented cafeteria diet ameliorates glucose intolerance in rats

**DOI:** 10.1002/fsn3.3603

**Published:** 2023-08-06

**Authors:** Hatice Ozcaliskan Ilkay, Derya Karabulut, Gonca Kamaci Ozocak, Ecmel Mehmetbeyoglu, Emin Kaymak, Betul Kisioglu, Betul Cicek, Asli Akyol

**Affiliations:** ^1^ Faculty of Health Sciences, Department of Nutrition and Dietetics Hacettepe University Ankara Turkey; ^2^ Faculty of Health Sciences, Department of Nutrition and Dietetics Erciyes University Kayseri Turkey; ^3^ Faculty of Medicine, Department of Histology and Embryology Erciyes University Kayseri Turkey; ^4^ Faculty of Veterinary Medicine, Department of Laboratory Animals Science Erciyes University Kayseri Turkey; ^5^ Erciyes University Betul Ziya Eren Genome and Stem Cell Center Kayseri Turkey; ^6^ Faculty of Medicine, Department of Histology and Embryology Yozgat Bozok University Yozgat Turkey

**Keywords:** cafeteria diet, glucose tolerance, obesity, quinoa, rats

## Abstract

Quinoa (*Chenopodium quinoa* Willd.) is a pseudocereal with rich nutritional composition, gluten free, and organoleptic. The primary aim of this study was to elucidate the possible protective roles of quinoa in glucose homeostasis in a model of cafeteria diet‐induced obesity. Male Wistar rats (3 weeks of age) were randomly allocated to be fed by; control chow (CON; *n* = 6), quinoa (QUI; *n* = 6), cafeteria (CAF; *n* = 6), or quinoa and cafeteria (CAFQ; *n* = 6) for 15 weeks. CAFQ resulted in decreased saturated fat, sugar, and sodium intake in comparison with CAF. Compared to CON, CAF increased body weight gain, plasma insulin, plasma glucose, decreased liver IRS‐1, AMPK mRNA expressions, and pancreatic β‐cell insulin immunoreactivity, and developed hepatocyte degeneration and microvesicular steatosis. Compared to CAF, QUI lowered body weight, plasma glucose, and plasma insulin, increased liver IRS‐1 and AMPK mRNA expressions, and pancreatic β‐cell insulin immunoreactivity. Compared to CAF, CAFQ lowered plasma glucose, increased liver IRS‐1 mRNA expressions, increased pancreatic β‐cell insulin immunoreactivity, and lowered hepatocyte degeneration and microvesicular steatosis. Dietary treatments did not influence IRS‐2, AKT2, and INSR mRNA expressions. HOMA‐IR, HOMA‐β, and QUICKI were also similar between groups. Restoration of insulin in CAFQ islets was as well as that of CON and QUI groups. In conclusion, as a functional food, quinoa may be useful in the prevention of obesity and associated metabolic outcomes such as glucose intolerance, disrupted pancreatic β‐cell function, hepatic insulin resistance, and lipid accumulation.

## Graphical abstract

The pseudocereal quinoa (*Chenopodium quinoa* Willd.) diet decreased plasma glucose and insulin levels and increased insulin sensitivity in the liver. The cafeteria diet resulted in increased body weight, glucose intolerance, impaired hepatic insulin signaling, microvesicular steatosis, and reduced β‐cell insulin immunoreactivity. Quinoa added to the cafeteria diet reduced body weight and improved glucose intolerance, hepatic insulin signaling, and microvesicular steatosis.

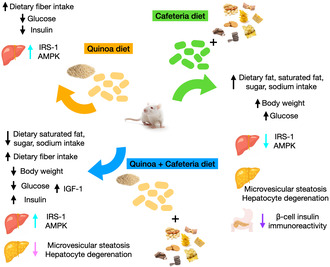

## 1 INTRODUCTION

Obesity is a major health problem around the world. Rates of obesity have nearly tripled since 1975 according to World Health Organization's global obesity data (WHO, 2021). The need for efficient strategies in preventing obesity becomes evident since obesity and its complications exert detrimental effects on the physiological, economic, and social aspects of life (Boutari & Mantzoros, 2022). Sedentary lifestyle and consumption of unbalanced, energy‐dense diets are the major causes of obesity (Hruby et al., 2016). Studies have shown that medical nutritional therapy for obesity that consists of lower energy intake, well‐balanced and ‘healthy’ diet throughout life could be difficult to maintain in some cases (Gupta, 2014; Löffler et al., 2021). However, this situation could be helped by consuming functional foods, which have low energy content but high protein, fiber, and bioactive compounds (Esmaeili et al., 2022). Recently, some functional foods and their bioactive food components have attracted attention for their impact upon appetite suppression and satiety and prolongation (Rebello et al., 2014).

Quinoa is an Andean origin pseudocereal, is known as the “mother of all grains”, and considered as a “golden grain” by civilizations in ancient times (Filho et al., 2017). Quinoa is a popular food, due to its rich nutritional composition, gluten‐free properties, and organoleptic compatible features with foods and beverages. It provides all essential amino acids and its protein content is higher than rice and whole wheat (Mithila & Khanum, 2015). Beyond being an excellent plant‐based protein source in the human diet, it contains high levels of unsaturated fatty acids, fiber, vitamins, and minerals. It is reported that quinoa contains a variety of polysaccharides with the total fiber content reaching up to 8.52%. Calcium, phosphorus, and iron content of quinoa is also higher than rice and wheat (Wang et al., 2023). In addition, quinoa has several bioactive components such as flavonoids (quercetin and kaempferol) and isoflavones (daidzein and genistein) (Vega‐Gálvez et al., 2010). Further, studies showed that quinoa exhibited one of the highest antioxidant activities among grains and pseudocereals (FAO & CIRAD, 2015).

Quinoa's great potential to serve as a nutritious dietary component has led to increasing interest from researchers looking into novel foods and preventing food insecurity (Chaudhary et al., 2023). Studies on rodents have shown decreased lipid peroxidation and increased antioxidant capacity (Pasko et al., 2010a; Paśko et al., 2010b), lower food intake (Lopes et al., 2019; Mithila & Khanum, 2015), lower blood glucose and lipids levels, lower accumulation of adipose tissue (Lopes et al., 2019), reduced plasma total cholesterol and LDL cholesterol (Fotschki et al., 2020) and ameliorated hyperlipidemia (Cao et al., 2020) by different quinoa supplemented diets. Further, the protective effects of quinoa supplementation were investigated along with different dietary models used to induce obesity in rodents (Cao et al., 2020; Pasko et al., 2010a; Paśko et al., 2010b).

The cafeteria diet is used to trigger diet‐induced obesity in laboratory animals by introducing highly energetic and palatable human foods along with the chow diet (Sclafani & Springer, 1976), thus increasing appetite and inducing hyperphagia by the significantly increased amounts of dietary fat, sugar, and salt intakes (Oliva et al., 2017). Studies using animal models of obesity have proposed that the cafeteria diet mimics the Western diet with its highly palatable foods contributing to weight gain, obesity, and associated metabolic alterations (Akyol et al., 2009; Buyukdere et al., 2019; Kabasakal Çetin et al., 2021; Lalanza & Snoeren, 2021). In addition, the cafeteria diet‐induced obesity phenotype is proposed to be closely aligned with human obesity than diets consisting of purified nutrient sources (Johnson et al., 2016; Martire et al., 2014). However, none of the studies have examined whether quinoa supplementation can reverse adverse effects of cafeteria diet on glucose homeostasis‐related metabolic parameters. Therefore, the primary aim of this study was to elucidate the possible protective role of quinoa in glucose homeostasis in a rat model of cafeteria diet‐induced obesity.

## 2 MATERIALS AND METHODS

### 2.1 Animals and dietary intervention

All experiments were performed in accordance with Directive 2010/63/EU under the license from the Ethics Committee of Erciyes University, Kayseri, Turkey, number: 18/007 and checks ARRIVE guidelines. All animals were housed individually in plastic cages and subjected to a 12‐h light–dark cycle (08.00 and 20.00) at a temperature of 23 ± 3°C and 45% humidity. The animals had ad libitum access to food and water at all times. After 1 week of habituation (standard chow and drinking water), male Wistar rats (3 weeks of age) were randomly allocated to be fed by one of the following four diets; control chow diet (CON; *n* = 6), quinoa diet (QUI; *n* = 6), cafeteria diet (CAF; *n* = 6), or quinoa and cafeteria diet (CAFQ; *n* = 6) for 15 weeks.

The CON diet met the nutritional content of D10012G purified rodent diet (Reeves et al., 1993), whereas the QUI diet consisted of white quinoa (315 g quinoa/1000 g diet) that was replaced the 50% of cornstarch, maltodextrin, and sucrose content of D10012G as previously described (Paśko et al., 2010). The CAF group received the cafeteria diet that included 10 highly palatable and energetic human foods such as peanuts, cheese, potato chips, variety of biscuits, corn chips, crackers, and variety of chocolates along with D10012G purified rodent diet. Five of the cafeteria diet foods were provided daily in excess quantities on the cage floor and were changed daily by replacing three of them with novel foods to maintain a variety. Therefore, the rats did not receive the same foods for more than two consecutive days. The CAFQ group received a cafeteria diet and a QUI diet. All of the chow diets and foods of the cafeteria diets were individually weighed daily at a specific time point (09.00–10.00 am) (Akyol et al., 2009; Buyukdere et al., 2019; Kabasakal Çetin et al., 2021). The diets used in this study were purchased from ARDEN Research & Experiment, Ankara and were prepared following the OpenSource Diets (Research Diets Inc.).

During the dietary intervention, the daily intake of nutrients were calculated from the manufacturers' data (Table 1). At the end of the dietary intervention, an intraperitoneal glucose tolerance test (IPGTT) was performed, and all animals were culled using CO_2_ asphyxia and cervical dislocation. Blood samples were taken by cardiac puncture, and major organs were weighed and snap‐frozen in liquid N_2_ and stored for further analysis.

**TABLE 1:** Nutritional composition of the diets.

Energy and nutrients	CON	QUI	CAF foods
Carbohydrate (% energy)	63.9	54.6	42.9
Protein (% energy)	20.3	25.5	7.8
Fat (% energy)	15.8	19.8	49.2
Energy (kcal/g)	3.9	3.9	4.8
Saturated fat (g/100 g)	1	0.6	11.8
Sugar (g/100 g)	10	6.3	21.2
Fiber (g/100 g)	5	8.6	3.2
Sodium (mg/100 g)	316	230	364

Abbreviations: CAF, cafeteria diet; CON, control chow diet; QUI, quinoa chow diet.

### 2.2 Intraperitoneal glucose tolerance test

At the end of the 15‐week dietary intervention, all animals were subject to an IPGTT after 12 hours of night fasting (Marineli Rda et al., 2015). At the beginning of the test, the rats were restrained to obtain a baseline blood sample from the superficial tail vein, under local anesthesia. After baseline sampling, 1 mL/100 g body weight glucose (20 g/100 mL in 0.9% saline) was administered via intraperitoneal injection (overall dose of 2‐g glucose/kg body weight). Blood was sampled from the tail vein at 30‐, 60‐, 90‐, and 120‐min postglucose administration, and blood glucose levels were measured by a glucometer (ACCU‐CHEK Active, Roche Diabetes Care). After glucose sampling, all blood samples were collected into heparinized capillary tubes and centrifuged (hematocrit centrifuge) for plasma collection. The plasma was stored at –80°C until further analysis.

Plasma insulin levels were measured by a commercially available ELISA kit (Shanghai Sunred Biological Technology Co., Ltd) from the samples obtained at 0, 30, and 60 min (Ayala et al., 2010; Bowe et al., 2014). Data on the area under the curve (AUC) for glucose were obtained using GraphPad Prism version 5 (Graphpad Software Inc.).

Fasting plasma glucose and insulin concentrations were used to measure three indexes that are: the homeostasis model assessment‐insulin resistance (HOMA‐IR) index, the quantitative insulin sensitivity check index (QUICKI), and homeostatic model assessment of β‐cell function (HOMA‐β). The equations were as follows:

HOMA‐IR = fasting insulin (μU/mL) × fasting glucose (mg/L)/405 (Buyukdere et al., 2019), QUICKI = 1/[log(fasting insulin (μU/mL)) + log (fasting glucose (mg/dL))] (Yuruk & Nergiz‐Unal, 2017),

HOMA‐β = [20 × (fasting insulin (μIU/mL))]/[fasting glucose (mmol/mL‐3.5)] (Matthews et al., 1985).

A final blood sample (120 min after administration of glucose) was taken by cardiac puncture. Further, commercially available ELISA kits (Shanghai Sunred Biological Technology Co.) were used to measure plasma levels of insulin‐like growth factor‐1 (IGF‐1) and glucagon‐like peptide‐1 (GLP‐1).

### 2.3 RNA isolation and real‐time PCR

Total RNA was extracted from the liver using QIAzol Lysis Reagent (QIAGEN) according to the manufacturer's protocol. The quantity and quality of isolated RNA were measured using NanoDrop 2000 Spectrophotometer (Thermo Scientific). Following isolation, RNA samples were stored at –80°C for further use in expression studies. The total RNA was converted to first‐strand complementary DNA using the RT2 First Strand Kit (QIAGEN), and incubated at 42°C for 15 min, with a next step of 5 min at 95°C. Quantitative real‐time PCR was used to measure mRNA expression levels of IRS1, IRS2, AKT2, AMPK, and INSR genes using SYBR Green I Master Kit (Roche, Swiss). PCR amplification was carried out using LightCycler® 480 II Real‐Time PCR System (Roche, Swiss). Cycling conditions were 95°C for 10 min, followed by 45 cycles of 95°C for 15 s, and 60°C for 1 min. All samples were studied in duplicate. ACTB (β‐actin) was used as the housekeeping gene. The relative expression of each mRNA was determined by the 2^‐ΔΔCt^ method as previously described and normalized using housekeeping gene (Schmittgen & Livak, 2008).

### 2.4 Histological procedure

At the end of the experiment, the liver and pancreatic tissues of rats were removed and detected in 4% formaldehyde solution. Standard histopathological techniques were applied to the liver and pancreatic tissues waiting in fixation solutions. Then tissue sections from the organs were taken and embedded in paraffin following dehydration (50%, 70%, 80%, 96%, three times absolute alcohol) and clearing (xylene). Five‐micrometer‐thick paraffin‐embedded liver sections of all rats were stained with hematoxylin–eosin (H&E) and periodic acid–Schiff (PAS) for a histological assessment as previously described. Digital photomicrographs of stained tissues were captured at 200× magnification using a light microscope (Olympus BX51) (Sayan et al., 2020). Hepatocyte degeneration, microvesicular steatosis, lobular infiltration, and portal infiltration parameters were evaluated in H&E‐stained liver sections. Glycogen storage was evaluated in PAS‐stained liver sections. All histological examinations of stained sections were evaluated by two blinded histologists. The evaluation score in liver sections after staining is as follows: negative (0), mild (1), moderate (2), and dense (3) levels (Sayan et al., 2020).

### 2.5 Immunohistochemistry

Pancreatic islets of Langerhans were visualized by performing immunohistochemistry with insulin primary antibody (sc‐8033, Santa Cruz Biotechnology). The pancreatic sections kept at 60°C overnight were washed in phosphate‐buffered saline (PBS) by passing through a series of xylene and then graded alcohol. It was then treated with a 5% citrate buffer for antigen recovery. Hydrogen peroxide (3%) was applied to the sections washed in PBS. For the next steps, staining kit (Thermo Scientific™, Lab Vision™, UltraVision™, Large Volume Detection System: anti‐Polyvalent, HRP, TP‐125‐HL) was used. After treatment with diaminobenzidine (Thermo Scientific™, DAB Plus Substrate System, TA‐125‐HDX) to make immune reactivities visible, they were washed with deionized H₂O. Counterstained sections with Gill hematoxylin were washed. Finally, the sections passed through xylene by removing the water with increasing alcohol series were covered with a sealing medium (Entellan®, Merck) and examined under a light microscope (Olympus BX51). Morphometric measurements of immunoreactivity intensity of insulin in ß‐cells from the digitalized images of sections were carried out using the ImageJ program. Data were obtained from one pancreas section from each animal (24 animals/4 groups), and a total of 45 islets were analyzed per group (Karabulut & Sonmez, 2021).

### 2.6 Statistical analysis

GraphPad\Prism version 7 was used for statistical analysis. Power analysis indicated that six animals per group were sufficient to detect a minimum 12% energy intake difference with a power of 80% and *α* of .05. The effect of diet on metabolic parameters that were normally distributed was examined using one‐way ANOVA, whereas the change over time of the longitudinal data (for instance, weekly body weights or nutrient intakes) was examined using repeated‐measures analysis. Values are expressed as mean with their standard errors. *p* < .05 was considered statistically significant. Post‐hoc testing (Tukey's test) was applied to define the main effects of the diets (Akyol et al., 2009).

## 3 RESULTS

### 3.1 Body weight changes and nutrient intakes

**FIGURE 1:**
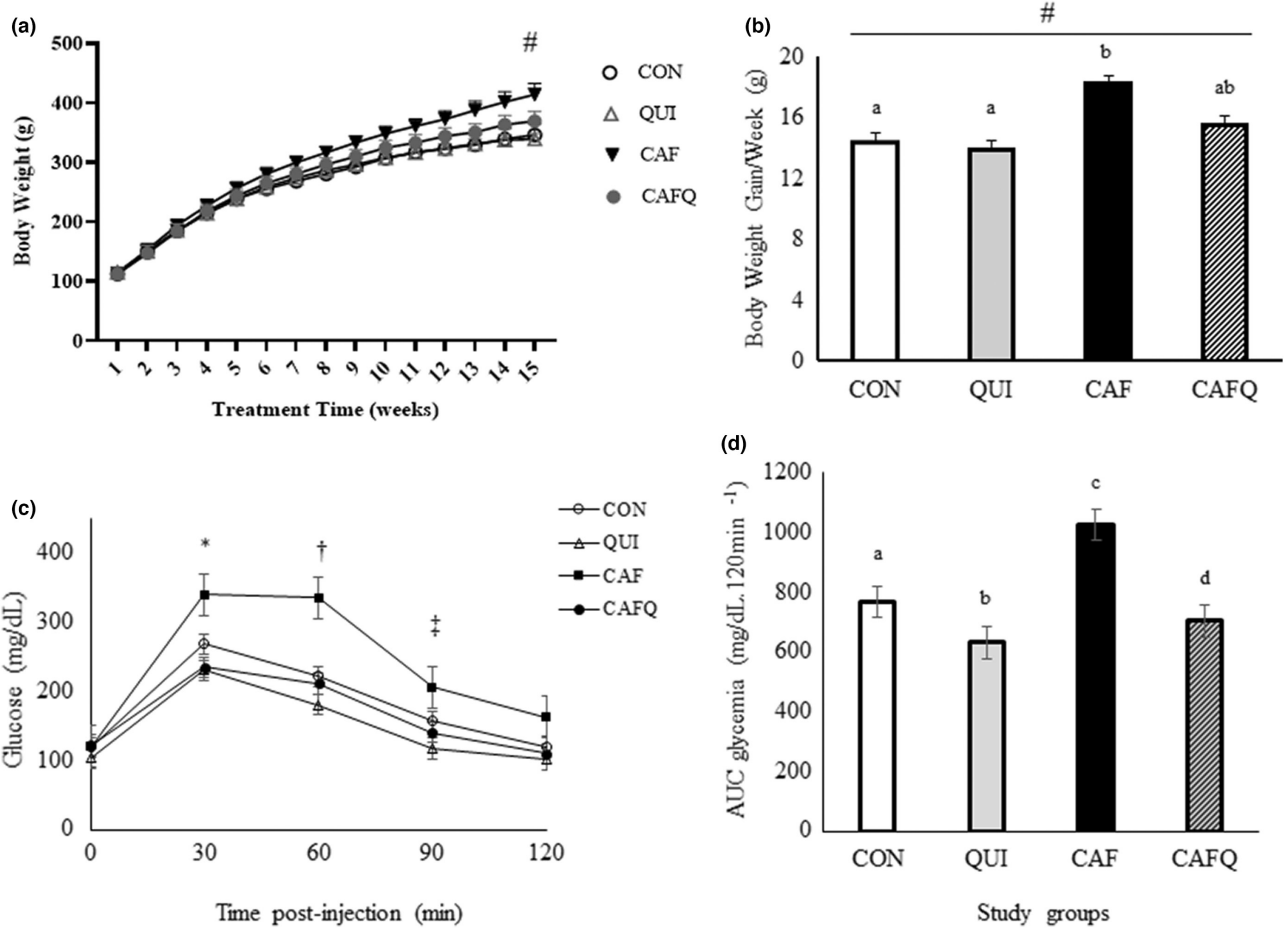
Body weight changes and glycemic response of the diets. (a) Body weight changes during the study weeks in rats fed different diets. (b) Average body weight gain per week in rats fed different diets. (c) Glucose tolerance test in rats. (d) Area under curve data of glycemic response after glucose tolerance test. Values are represented as mean ± SEM. ^#^Repeated measures ANOVA indicated significantly different values between groups over weeks (A: diet and treatment time interaction, *p* < .001, B: diet [*p* = .014], treatment time [*p* < .001], and interaction between diet and treatment time [*p* = .028]). *Lower in QUI and CAFQ compared to CAF at 30 min postinjection (*p* < .05). ^†^Lower in QUI and CAFQ compared to CAF at 60 min postinjection (*p* < .05). ^‡^Lower in QUI compared to CAF at 60 min postinjection (*p* < .05). Different letters indicate significant differences between groups (*p* < .001). CAF, cafeteria diet; CAFQ, quinoa and cafeteria diet; CON, control chow diet; QUI, quinoa diet.

All rats had similar body weight at the beginning of the study and gained weight during the treatment weeks. Although the average body weights were similar between the groups (CON, 264.14 ± 8.01 g; QUI, 265.13 ± 7.58 g; CAF, 297.47 ± 9.80 g; CAFQ, 276.30 ± 8.75 g), a significant interaction between diet and treatment time indicated that body weight changes were different over weeks (*p* < .001) (Figure 1a). When average body weight gain per week was analyzed, it appeared that diet (*p* = .014), treatment time (*p* < .001), and interaction between diet and treatment time (*p* = .028) were statistically significant (Figure 1b). The final body weights of the rats among the groups CON (346.30 ± 18.28 g) and QUI (339.67 ± 8.73 g) were significantly lower than CAF (416 ± 18.61 g), whereas CAFQ (370.17 ± 16.31 g) displayed a similar body weight to CON and QUI (Figure 1a). The body weight gain per week of the CAF (18.24 ± 1.03 g/week) was increased in comparison with those fed the CON (14.44 ± 1.08 g/week) and QUI (13.92 ± 1.07 g/week). The body weight gain per week of CAFQ (15.54 ± 1.09 g/week) was similar to the other groups (Figure 1b).

Table 2 shows the average daily energy and nutrient intakes of the diet groups during the study. The CON, QUI, CAF, and CAFQ diets significantly influenced all components of nutritional intakes (*p* < .05). While rats fed the CAF had similar energy intake to CON, rats fed the CAFQ had lower energy intake than CON. Carbohydrate intake was significantly lower in QUI, CAF, and CAFQ than in CON. CAF and CAFQ had significantly lower protein intakes in comparison with CON and QUI. The impact of diet on fat intake of the animals was apparent as rats fed the CAF and CAFQ had significantly increased fat intake in comparison with those fed the CON and QUI. At this point, fat intake of CAF and CAFQ groups was similar. The different nutritional composition of QUI diet was reflected in nutritional intakes of animals in this group as their fat and protein intakes were significantly higher than CON. CAF group had the highest intake of saturated fatty acids, sugar, and sodium, whereas these nutrient intakes decreased in rats fed CAFQ diet. Fiber intake of CON and CAFQ was similar, whereas CAF had lower, and QUI had higher fiber intakes (Table 2).

**TABLE 2:** Average daily intake of feed, energy, and nutrients.

Dietary component	CON	QUI	CAF	CAFQ
	(*n* = 6)	(*n* = 6)	(*n* = 6)	(*n* = 6)
Energy intake (kcal/day)*	99.57 ± 1.37^**a**^	106.80 ± 1.18^**b**^	96.31 ± 1.28 ^**a**^	88.20 ± 1.17^**c**^
Carbohydrate intake (g/day)*	16.09 ± 0.22^**a**^	14.78 ± 0.1^**b**^	12.26 ± 0.22^**c**^	10.22 ± 0.17^**d**^
Protein intake (g/day)*	5.03 ± 0.07^**a**^	6.47 ± 0.15^**b**^	3.66 ± 0.06^**c**^	3.89 ± 0.08^**c**^
Fat intake (g/day)*	1.76 ± 0.02^**a**^	2.39 ± 0.03^**b**^	3.61 ± 0.04^**c**^	3.53 ± 0.05^**c**^
Saturated fatty acids intake (g/day)*	0.25 ± 0.01^**a**^	0.16 ± 0.01^**b**^	1.50 ± 0.02^**c**^	1.33 ± 0.03^**d**^
Sugar intake (g/day)*	2.51 ± 0.03^**a**^	1.63 ± 0.02^**b**^	3.48 ± 0.09^**c**^	2.64 ± 0.07^**a**^
Fiber intake (g/day)*	1.26 ± 0.02^**a**^	2.36 ± 0.03^**b**^	0.86 ± 0.02^**c**^	1.19 ± 0.03^**a**^
Sodium intake (mg/day)*	79.45 ± 1.09^**a**^	68.50 ± 1.62^**b**^	87.00 ± 1.49^**c**^	68.49 ± 1.47^**b**^

*Note*: Mean values with standard errors. Different letters indicate significant differences between groups. Abbreviations: CAF, cafeteria diet; CAFQ, quinoa and cafeteria diet; CON, control chow diet; QUI, quinoa diet.

* ANOVA indicated that diet (*p* < .05), treatment time (*p* < .001), and diet*treatment time interaction (*p* < .001) significantly influenced the dietary component.

### 3.2 Plasma insulin concentrations and glucose homeostasis

Baseline plasma glucose levels were similar between the groups. Following the IPGTT, plasma glucose levels were significantly different between the groups at 30, 60, and 90 min (effect of diet *p* = .008, time *p* < .001, and diet*time interaction *p* = .011). Post‐hoc analysis showed that plasma glucose levels were significantly lower in QUI and CAFQ compared to CAF at 30 min postinjection (*p* < .05). A similar pattern was also observed at 60 min postinjection (*p* < .05). At 90 min postinjection, plasma glucose levels were significantly lower in QUI compared to CAF (*p* < .05) and all groups displayed comparable levels at 120 min postinjection (Figure 1c). The differences in plasma glucose levels were also reflected in AUC. QUI and CAFQ had significantly reduced, whereas CAF had significantly increased AUC of glucose in comparison with CON (*p* < .05). In addition, the AUC of glucose was significantly higher in CAFQ than in QUI (*p* < .05) (Figure 1d).

Baseline insulin levels were similar between the groups. It appeared that the plasma insulin levels at 30 and 60 min postinjection were significantly different between the groups. At 30 min, insulin levels of QUI were significantly lower than CON, CAF, and CAFQ (*p* < .05) and the groups CON, CAF, and CAFQ did not exhibit a further difference. At 60 min, CAFQ group had significantly higher insulin levels than CAF. Despite the differential effects of dietary treatment on glucose and insulin levels of the study groups, other parameters in plasma related to glucose homeostasis such as HOMA‐IR, HOMA‐β, and QUICKI were similar between groups. Further, plasma levels of GLP‐1 were found to be similar, whereas plasma IGF‐1 levels showed significantly different among the diet groups. CAFQ exhibited significantly higher IGF‐1 levels than QUI and CAF (Table 3).

**TABLE 3:** Insulin concentrations and glucose homeostasis‐related blood parameters.

Parameter	CON (*n* = 6)	QUI (*n* = 6)	CAF (*n* = 6)	CAFQ (*n* = 6)
Baseline insulin (μIU/mL)	11.60 ± 0.22	10.75 ± 0.64	11.56 ± 0.26	10.79 ± 0.40
30‐min insulin (μIU/mL)*	13.85 ± 0.62^a^	10.77 ± 0.54^b^	12.82 ± 0.31^a^	13.08 ± 0.44^a^
60‐min insulin (μIU/mL)*	13.73 ± 0.30^ab^	12.63 ± 0.30^ab^	12.50 ± 0.34^b^	13.78 ± 0.34^a^
HOMA‐IR	3.42 ± 0.18	2.76 ± 0.26	3.44 ± 0.05	3.27 ± 0.28
HOMA‐β	77.14 ± 6.33	100.28 ± 9.14	73.18 ± 4.29	72.18 ± 11.09
QUICKI	0.32 ± 0.002	0.33 ± 0.004	0.32 ± 0.001	0.32 ± 0.004
IGF‐1 (ng/mL)	157.89 ± 6.97^ab^	145.28 ± 5.63^a^	137.03 ± 6.62^a^	173.15 ± 5.24^b^
GLP‐1 (pg/mL)	12.51 ± 0.88	10.26 ± 0.26	10.68 ± 0.86	10.69 ± 0.71

*Note*: Mean values with standard errors. Different letters indicate significant differences between groups. Abbreviations: CON, control chow diet; CAF, cafeteria diet; CAFQ, cafeteria diet with quinoa chow diet HOMA‐IR, homeostasis model assessment‐insulin resistance; HOMA‐β, homeostatic model assessment of β‐cell function; IGF‐1, insulin‐like growth factor‐1; QUI, quinoa chow diet; QUICKI, the quantitative insulin sensitivity check index.

* ANOVA indicated that diet (*p* < .05) significantly influenced the insulin levels at 30 and 60 min postinjection.

### 3.3 Insulin signaling and histology of the liver

**FIGURE 2:**
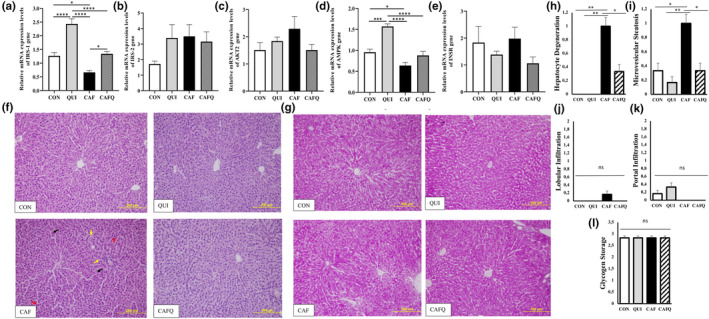
Relative mRNA expression levels of hepatic genes involved in the insulin signaling pathway and hepatic histological analysis. Relative mRNAs expression levels of hepatic (a) IRS‐1 (b) IRS‐2 (c) AKT2 (d) AMPK (e) INSR genes. (f) Representative photomicrographs for liver section of the dietary groups with H&E. (g) Representative photomicrographs for liver section of the dietary groups with PAS staining. (h) Hepatocyte degeneration score. (i) Microvesicular steatosis score. (j) Lobular infiltration score. (k) Portal infiltration score. (l) Glycogen storage score. *p < .05; **p < .01; ***p < .001; ****p < .0001. Black arrows; hepatocyte degeneration, red arrows; acidophilic hepatocyte, yellow arrows; microvesicular steatosis. Scale bar: 200 μm. CAF, cafeteria diet; CAFQ, quinoa and cafeteria diet; CON, control chow diet; QUI, quinoa diet.

To investigate the basis of glucose intolerance in animals exposed to cafeteria diet, we examined the mRNA expression of five components of the insulin signaling pathway in the liver. The dietary models used in this study significantly affected the expression of insulin receptor substrate (IRS‐1) and AMP‐activated protein kinase (AMPK), whereas IRS‐2, RAC‐beta serine/threonine‐*protein kinase* (AKT2), and insulin receptor (INSR) were unaffected. The IRS‐1 expression was significantly upregulated in QUI (*p* < .001) and downregulated in CAF (*p* < .05) when compared to CON. The addition of quinoa in cafeteria diet exerted a normalization in the expression of IRS‐1 since CAFQ had similar expression level of IRS‐1 in comparison with CON (*p* > .05). Among the rats exposed to QUI diet, the expression of AMPK was significantly higher than CON (*p* < .001). Although CAFQ exhibited a similar expression of AMPK in comparison with CAF (*p* > .05), the levels were also like CON, whereas CAF displayed significantly lower expression of AMPK than CON (*p* < .05) (Figure 2a–e).

The CON group exhibited normal liver architecture with regular hepatocyte cell cords organized radially from the vena centralis. Moreover, regularly distributed sinusoids were observed between the cell cords, whereas the portal areas had also a regular arrangement. The nuclei of the hepatocytes were clearly observed. Hepatocyte cell cords, sinusoid localization, and portal areas appeared to be normal. Similar results were obtained from histological analysis of liver sections of QUI group. As compared to CON group, exposure to CAF diet induced microvesicular steatosis, which was observed in hepatocytes close to some portal areas. In CAF group, partial degeneration in hepatocyte cell nuclei and more acidophilia staining in some hepatocytes was remarkable. In addition, dispersion of some cell cords radially extending from the vena centralis in some areas of the hepatic tissues and narrowing of the sinusoids was observed. Supplementation with quinoa in CAF diet‐fed rats substantially alleviated microvesicular steatosis. In the CAFQ group, hepatocyte degeneration was slighter than that in CAF group (Figure 2f,h,i). No appreciable lobular or portal infiltration was observed in all groups (Figure 2f,j,k). Representative micrographs of PAS at the end of experiment displayed densely glycogen deposits in cytoplasm of all hepatocytes (Figure 2g,l).

### 3.4 Insulin immunoreactivity in pancreatic ß‐cells

**FIGURE 3:**
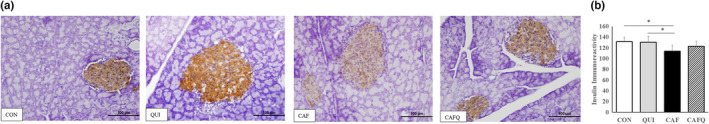
Analysis of ß‐cells in pancreatic tissue. (a) Immunohistochemical staining results of insulin immunoreactivity of ß‐cells in pancreatic tissue of all diet groups. (b) Insulin immunoreactivity score. Scale bar: 100 μm. *p < .05. CAF, cafeteria diet; CAFQ, quinoa and cafeteria diet; CON, control chow diet; QUI, quinoa diet.

The CON group showed strong immunoreactivity of insulin in ß‐cells in the form of dark brown granules which occupy most of the islet. Immunostained ß‐cells from CON group appeared to be similar to that of QUI group. As compared with CON and QUI groups, a noteworthy reduction in the immunohistochemical expression of insulin in ß‐cells was observed in pancreatic section of a CAF group. Insulin immunoreactivity was also observed in the cytoplasm of beta cells in pancreatic islets belonging to the CAFQ group (Figure 3a,b).

Morphometric analysis revealed that insulin expression in QUI group was similar to that of CON group as evidenced by photomicrographs of insulin immunohistochemical staining of pancreatic islets. Furthermore, immunoreactivity density in CAF group significantly decreased in comparison with CON and QUI groups. Immunoreactivity density tended to increase in CAFQ group compared to the CAF group, but the difference was not statistically significant. Moreover, restoration of insulin in CAFQ islets was as well as that of CON and QUI groups (Figure 3a,b).

## 4 DISCUSSION

Cafeteria diet induces a phenotype of obesity with glucose intolerance and inflammation as a model of human metabolic syndrome (Sampey et al., 2011) and human obesity (Buyukdere et al., 2019; Sampey et al., 2011). The present study showed that CAF increases body weight. Current literature indicates that cafeteria diet‐fed rats have also shown increased body weight (Buyukdere et al., 2019; Johnson et al., 2016; Kabasakal Çetin et al., 2021; Sampey et al., 2011) and body weight fat percentage due to increased energy, fat, and carbohydrate intake (Buyukdere et al., 2019) indicating obesity. The increased food intake may also be held responsible for the developed obesity as cafeteria diet was reported to induce hyperphagia (Buyukdere et al., 2019; Sampey et al., 2011). This may be due to the high glycemic index (GI) foods that may lead to increased hunger in the middle of the postprandial period due to insulin‐induced hypoglycemia thus, causing prolonged and persistent hyperphagia (Lopes et al., 2019). Moreover, CAF elevated glucose levels after IP‐GTT, which may indicate glucose intolerance. Similarly, studies have shown hyperinsulinemia, hyperglycemia, glucose intolerance (Sampey et al., 2011), and less insulin sensitivity (Sampey et al., 2011). In contrast, despite insulin resistance and high insulin levels, cafeteria feeding did not change the insulin‐mediated drop in glucose (Sampey et al., 2011) as some glucose homeostasis parameters did not change in CAF. However, increased IGF‐1 in CAFQ may be related to insulin resistance (Franco et al., 2006) induced by cafeteria diet (Sampey et al., 2011). Hyperglycemia in CAF may possibly be dependent on peripheral insulin resistance or impaired glucose uptake of tissues.

Further, studies using the cafeteria diet model in rats showed increased fat accumulation (Maeda Júnior et al., 2018), microvesicular steatosis, and hepatosteatosis in liver tissues, indicating the ability of the diet to promote nonalcoholic steatohepatitis (Sampey et al., 2011). Hepatosteatosis is linked with visceral adiposity (Maeda Júnior et al., 2018; Sampey et al., 2011), and the high GI of the diet followed by greater incorporation of glucose into lipids may be associated with increased fat deposition (Lopes et al., 2019). Moreover, the fatty liver induced by a cafeteria diet in rats led to the overstimulation of hepatic gluconeogenesis in an environment with glucagon and fatty acids substrates, which was proposed to be the reason of hepatic insulin resistance (Maeda Júnior et al., 2018; Sampey et al., 2011). Further, the lower hepatic expression of IRS‐1 and AMPK due to CAF may be associated with the presence of insulin resistance (Glass & Olefsky, 2012) and hepatic lipid accumulation (Lanaspa et al., 2012). Likely, the cafeteria diet has shown increased hepatic gene expression of IRS‐1 but not INSR along with greater activation of the AMPK pathway indicating an increase in fat synthesis and accumulation (Castro et al., 2015). Additionally, impaired insulin sensitivity is associated with dysfunction of the pancreas islets (Sampey et al., 2011) and slightly hypertrophic and disfigured pancreatic islets with dramatically distorted architecture in cafeteria‐fed rats were shown (Sampey et al., 2011). An inverse relationship between quinoa supplementation and body weight gain has been reported previously, in studies using high‐fat diets as an obesity model (Cao et al., 2020; Song et al., 2021). Our results showed similar effects as CAFQ displayed an attenuated body weight in comparison with CAF. In fact, a potential factor involved in the attenuated body weight of CAFQ group appeared to be the decreased total energy intake due to lower ingestion of sugar, saturated fatty acids, and higher ingestion of fiber. It is apparent that these properties of the CAFQ diet originated from the low GI carbohydrate, high dietary fiber, quality protein, and n‐3 and n‐6 fatty acids content of quinoa. Rats consuming quinoa and amaranth supplemented diets exerted lower food intake than the control diet (casein); hence, these two pseudocereals exhibited very high potential to control appetite (Mithila & Khanum, 2015). Likewise, CAFQ decreased the total energy intake by 8% in comparison with CAF by modifying the cafeteria feeding to a diet higher in protein and lower in fat and simple carbohydrates. These results provide additional evidence to the findings associated with the suppression of energy intake and regulation of body weight by the regular consumption of pseudocereals, such as quinoa. The results of quinoa on body weight may be beneficial to prevent glucose intolerance shown to be induced by obesity (Buyukdere et al., 2019; Hruby et al., 2016; Yuruk & Nergiz‐Unal, 2017).

The main outcome of the current study was that QUI and CAFQ displayed attenuated plasma glucose levels which also indicates the regenerative influence of quinoa consumption in despite of cafeteria feeding. This antihyperglycemic effect of quinoa is in broad agreement with other studies that measured blood glucose levels following the consumption of quinoa‐supplemented diets (Mithila & Khanum, 2015), quinoa‐supplemented diets with high carbohydrate content (Lopes et al., 2019; Paśko et al., 2010), aqueous extract of quinoa (Azzane et al., 2022), and sprouted quinoa yoghurt in rodents (Obaroakpo et al., 2020). Accordingly, a clinical trial examining the influence of daily consumption of quinoa‐enriched bread, providing 20‐g quinoa flour, compared with a 100% refined wheat bread control reported that the cumulative AUC of blood glucose for the last 4 days of the quinoa intervention was significantly lower than the wheat treatment (Li et al., 2018). The antihyperglycemic effects of quinoa may be due to the maintenance of plasma‐free fatty acids from the slow release of glucose (low GI) leading to the protection of insulin‐mediated glucose uptake therefore insulin resistance (Lopes et al., 2019; Mithila & Khanum, 2015). Furthermore, delayed digestion and reduced carbohydrate absorption in particularly starch (low GI) (Li et al., 2018; Lopes et al., 2019) and tocopherols (Lopes et al., 2019; Paśko et al., 2010) or polyphenols contents of quinoa may also play a role (Li et al., 2018; Lopes et al., 2019; Paśko et al., 2010).

The antihyperglycemic effect of quinoa in CAFQ was independent from insulin levels as only the QUI group exhibited lower insulin levels at 30 min after injection. While the data on the AUC glucose indicated a recovered glucose intolerance in CAFQ, it was clear that differences were mainly a product of lower peak glucose concentrations, with no marked impairment of clearance at 2 h postglucose injection in CAF. Possibly, a mechanism associated with hyperglycemia is impaired glucose uptake of tissues. Similarly, a study on rats showed enhanced glucose uptake, hepatic glycogen synthesis, and liver glycogen while reducing gluconeogenesis as hypoglycemic effects of quinoa (Obaroakpo et al., 2020). Particularly, the liver insulin signaling provides novel data of increased IRS‐1 and AMPK gene expression of CAFQ and QUI, thus improving insulin signaling (Entezari et al., 2022; Glass & Olefsky, 2012). Likewise, increased liver AKT and AMPK mRNA levels were seen after sprouted quinoa yoghurt (Obaroakpo et al., 2020). Moreover, the reduced insulin resistance following quinoa supplementation was attributed to reduced levels of apoptotic, pro‐inflammatory (Erfidan et al., 2022), and lipid accumulation (Selma‐Gracia et al., 2021) gene expressions in the liver. Above all, excess glucose (Lopes et al., 2019) or insulin stimulation (Selma‐Gracia et al., 2021) may result in the conversion of glucose to triglycerides, thus increasing lipogenesis (Lopes et al., 2019). Quinoa showed mitigated microvesicular steatosis in livers of rats that may be associated with increased AMPK signaling (Obaroakpo et al., 2020).

A number of animal studies have verified the hepatoprotective potential of adding quinoa to energy‐dense diets (Ng & Wang, 2021) by reducing hepatic lipid accumulation (An et al., 2021; Song et al., 2021). Also, lower levels of lipid aggregation in the liver (Noratto et al., 2019), decreased hepatic fat (Gewehr et al., 2016) protected liver tissue structure (Obaroakpo et al., 2020) were reported in rodents due to quinoa (Noratto et al., 2019), quinoa flakes (Gewehr et al., 2016), or sprouted quinoa yoghurt (Obaroakpo et al., 2020). Concordant with previous studies, our study found that quinoa intervention alleviated CAF‐induced microvesicular steatosis and hepatocyte degeneration. The underlying mechanisms may be reduced hyperglycemia (Lopes et al., 2019), altered hepatic lipid metabolism genes (Song et al., 2021), anti‐inflammatory (Ng & Wang, 2021) and anti‐oxidant activities attributed to its abundant phytochemicals (saponins, phenolic acids, flavonoids, terpenoids, and steroids) (An et al., 2021; Song et al., 2021), lower blood cholesterol, and LDL‐C attributed to tocopherol and unsaturated fatty acid content (Gewehr et al., 2016). Additionally, it was suggested that quinoa's protein fraction inhibited reabsorption of bile acids in the small intestine and controlled cholesterol synthesis by suppressing of expression of hepatic HMG‐CoA reductase (Takao et al., 2005), its fiber content inhibited the absorption of dietary cholesterol (Noratto et al., 2019) and saponins in quinoa can inhibit pancreatic lipase which may lead to reduced cholesterol absorption (Navarro Del Hierro et al., 2021).

Further, pancreatic β‐cells were investigated since their failure to stimulate the secretion of insulin is seen in glucose intolerance (Hannon et al., 2018; Obaroakpo et al., 2020; Yang et al., 2016). β‐cell dysfunction is an important phase for the onset and progression of type 2 diabetes hence, with a predicted loss of approximately 50% of insulin secretion (Hannon et al., 2018; Yang et al., 2016). In the present study, pancreatic β‐cell insulin immunoreactivity supports the ability of quinoa to enhance glucose tolerance and insulin sensitivity and to favorably modulate the β‐cell function. This could be partially explained by the significant increase in the insulin level of the CAFQ compared to CAF at 60 min of IPGTT. Although there are no studies on quinoa and pancreatic insulin function, Obaroakpo et al. proposed that the ability of sprouted quinoa yoghurt to enhance glucose tolerance could be due to supporting pancreatic β‐cell function, which results in an increase in insulin secretion (Obaroakpo et al., 2020). In the absence of insulin sensitivity, enhanced β‐cell function plays a pivotal role in maintaining normal glucose tolerance (Cavaghan et al., 2000). Thus, quinoa supplementation may attenuate CAF‐induced decrease in insulin immunoreactivity of pancreatic islets.

In conclusion, this study successfully demonstrated that 315‐g quinoa seed/1000 g of chow diet supplementation to cafeteria diet reduced energy intake, differentiated macronutrient intake, regulated glucose homeostasis, attenuated microvesicular steatosis, hepatocyte degeneration, increased IRS‐1 and AMPK in the liver. In advance, potential attenuation of insulin immunoreactivity was seen in pancreatic β‐cells. Accordingly, this model will, therefore, be a suitable vehicle for future investigation of the potential antiobesity effects of functional foods in rats subjected to diet‐induced obesity protocols. Hence, our findings suggest that the cafeteria diet is an appropriate model to study human obesity and associated metabolic complications. On top of the potential antihyperglycemic effect of quinoa, future studies may focus on other mechanistic approaches related with glucose homeostasis such as inflammation, appetite signals, and the gut microbiome.

## AUTHOR CONTRIBUTIONS

**Hatice Ozcaliskan Ilkay:** Formal analysis (lead); investigation (lead); resources (lead). **Derya Karabulut:** Formal analysis (equal); methodology (equal); resources (equal). **Gonza Kamaci Ozocak:** Formal analysis (equal); methodology (equal); resources (equal). **Ecmel Mehmetbeyoglu:** Formal analysis (equal); methodology (equal); resources (equal). **Emin Kaymak:** Formal analysis (equal); methodology (equal); resources (equal). **Betul Kisioglu:** Data curation (equal); visualization (equal); writing – original draft (equal). **Betul Cicek:** Funding acquisition (lead); project administration (lead). **Asli Akyol:** Conceptualization (lead); methodology (lead); supervision (lead); writing – review and editing (lead).

## FUNDING INFORMATION

This work was supported by the Scientific Research Projects Coordination Unit of Erciyes University (No. TDK‐2018‐8222).

## CONFLICT OF INTEREST STATEMENT

The authors declare that they have no competing interests.

## ETHICS STATEMENT

This study was approved by the Erciyes University Local Ethics Committee for Animal Experiments (HADYEK), Kayseri, Turkey under the license from the number: 18/007.

## Data Availability

The data that support the findings of this study are available on reasonable request from the corresponding author.
